# Editorial: Quality of Life in Breast Cancer Patients and Survivors

**DOI:** 10.3389/fonc.2020.620574

**Published:** 2020-11-18

**Authors:** Marco Invernizzi, Jisun Kim, Nicola Fusco

**Affiliations:** ^1^ Physical and Rehabilitative Medicine, Department of Health Sciences, University of Eastern Piedmont, Novara, Italy; ^2^ Physical and Rehabilitative Medicine, University Hospital “Maggiore della Carità”, Novara, Italy; ^3^ Asan Medical Centre, Department of Surgery, College of Medicine, Ulsan University, Seoul, South Korea; ^4^ Division of Pathology, IEO, European Institute of Oncology IRCCS, Milan, Italy; ^5^ Department of Oncology and Hemato-Oncology, University of Milan, Milan, Italy

**Keywords:** breast cancer, quality of life, biomarkers, multidisciplinary management, survivorship

Every year more than 2 million women receive a new diagnosis of breast cancer worldwide (1). However, thanks to the increasing effectiveness of the screening programs and treatment protocols, the number of people who die of this disease has declined (Nardin et al.). Nowadays, caregivers are expected not only to prolong their patients’ life but also to preserve and improve their patients’ wellness before, during, and after the treatment (2). The continuum from initial diagnosis of cancer through the rest of the life (commonly referred to as “survivorship”) may evocate different issues and feelings to different subjects at different times (3). The ideal goal of survivorship is to return to, or even improve, the quality of life before diagnosis. This area incorporates a vast spectrum of concerns, such as treatment side effects, sexual life, pregnancy, social activities. In this complex scenario, a precision medicine approach is required to address quality of life issues and influence not only the decision-making but also treatment compliance.

We edited the present Research Topic to offer a new angle on some of the hot subjects about the quality of life in breast cancer survivors. Particular attention has been paid to the selection of articles focusing on patient-specific risk assessment methods and novel treatment strategies.

In a mini-review article, Nardin et al. present an overview of the long-term sequelae of breast cancer therapies. The Authors synthesize some of the most relevant aspects that may impact the quality of life after treatment, including cardiac toxicity related to anthracyclines, trastuzumab, endocrine therapy, and/or chest wall irradiation. In this respect, Axenie and Kurz give a particular emphasis on chemotherapy-induced sensorimotor alterations that may lead to polyneuropathy in a subset of patients. They provide insightful perspectives on the methods to assess these peripheral nervous system deficits and analyze the reliability of what they call “next-generation sensorimotor rehabilitation”. We believe that this article will shed fresh light on the use of cutting-edge digital technologies in breast cancer rehabilitation. A well-known unwanted effect of hormone therapy is osteoporosis (4). A nationwide retrospective cohort study on South Korean breast cancer survivors by Lee et al., supports the concept that selective estrogen receptor (ER) modulation with tamoxifen does not increase the risk of osteoporosis before menopause. Another original research article by Li et al. provides insights into the cumulative incidence and risk factors of second primary cancers after diagnosis and treatment of breast cancer. Specifically, they propose a nomogram for individual risk estimation, patient follow-up, and counseling in early-stage ER^NEG^ breast cancer survivors. The results of these studies highlight the significance of performing a tailored risk assessment strategy.

Multidisciplinarity is essential for the clinical management of breast cancer-related lymphedema (BCRL) (5, 6). This condition that frequently occurs after breast surgery and/or radiation therapy has tremendous implications on women’s quality of life (7). A comprehensive review article by Invernizzi, Lopez et al. summarizes the most clinically relevant steps of the lymphatic system ontogenesis to discuss molecular alterations that are likely involved in BCRL pathogenesis and progression. An original elaboration of publicly available genomic data is also provided, allowing for the discussion of the present and future perspectives in biomarker-based patients’ risk stratification. In a work by Vatansever et al., a local adaptation of the Upper Limb Lymphedema 27 (ULL-27) questionnaire has been studied. As not all patients can be assessed beyond their mother language, this work may help to implement this useful tool through a cross-cultural approach. Another important concern is represented by breast cancer fatigue (BCF), a condition characterized by persistent physical and/or mental stiffness (8). A prospective clinical study (Invernizzi, de Sire et al.) evaluated the feasibility and the effectiveness of a rehabilitation protocol for BCF, confirming how physical exercise may help improve the quality of life in breast cancer survivors. In line with these remarks, Chung et al. offer insightful viewpoints for the use of a mobile app-based approach to promote physical activity and decrease distress in breast cancer survivors. We would like to highlight that there are still not widely adopted guidelines to measure the muscular strength in these patients (9). For this reason, we have welcomed the study of Dos Santos et al., that evaluated the reliability and agreement for the 10-repetition maximum (10-RM) testing for upper (bench press) and lower (leg press) body strength. They observed that surgery may affect the upper limb but not lower limb strength in breast cancer patients. This paper is one of the few demonstrating that muscle strength increases during re-test, which may suggest significant effects either in technique improvement or muscle strength itself. Universal accessibility to rehabilitation/exercise enhancing centers is a subsequent problem to be considered.

During the past decade, literature embraced the concept that biology, body, and mind are vastly interconnected in both healthy and unhealthy individuals, including breast cancer patients (10). There are several lines of evidence that Tai chi, a Chinese martial art, may have a positive effect on both physical and psychological spheres in breast cancer survivors (11). A thorough update is provided in a systematic review and meta-analysis (Luo et al.) on the positive influence of this low-stress training method in improving the quality of life of breast cancer patients. Regrettably, many patients experience a decrease in cognitive and affective function after the diagnosis of breast cancer (12). This condition often relies on chemotherapy-related changes in brain structure and function (13). Because of its high vulnerability to adverse treatment effects, the hippocampus is becoming the focus of many lines of research (14). Here, Peukert et al. conducted a systematic review analysis of the current literature on hippocampus changes due to breast cancer treatment with subsequent cognitive and affective impairments. Surprisingly, they reveal that hippocampal alterations (e.g. volume loss, deformation, functional connectivity issues) may occur after all major types of treatment. These alterations are associated with cognitive impairments, where working memory, episodic memory, and prospective memory are the most frequently targeted affected domains. Disappointingly, cancer-related affective impairments are less studied and further research would be needed in this field. This is an area where multidisciplinary teamwork between oncologists and neurologist/psychologist is crucial.

Strategies to improve outcomes and subsequent quality of life should be put in place at the very beginning of the clinical workup of breast cancer patients. We believe that a biomarker-based approach in the risk assessment and treatment selection would represent a quantum leap for healthcare providers (15, 16). Based on their broad experience in the quality control of specimens and biomaterials handling in pathology labs, we invited the group of Berrino et al. to write a manuscript for this Research Topic. In their original research article, they provide previously unavailable data that monitored cold formalin fixation (4°C) is better than standard fixation for preserving DNA quality, which is pivotal in biomarkers assessment. An additional and little investigated type of treatment that may influence the outcome of breast cancer survivors is represented by neoadjuvant therapy (17). Chen et al. propose a nomogram based on machine learning that integrates magnetic resonance image data to select breast cancer patients that may benefit more from neoadjuvant chemotherapy. The final aim is the de-escalation of treatments that may impact the quality of life of breast cancer survivors. Beyond this ultimate goal for the physicians, breast cancer survivorship needs attention and efforts by society, communities, social media, and Institutions.

## Author Contributions

All authors contributed to the article and approved the submitted version.

## Conflict of Interest

The authors declare that the research was conducted in the absence of any commercial or financial relationships that could be construed as a potential conflict of interest.
